# Novel Sources of Biodiversity and Biomolecules from Bacteria Isolated from a High Middle Ages Soil Sample in Palermo (Sicily, Italy)

**DOI:** 10.1128/spectrum.04374-22

**Published:** 2023-04-18

**Authors:** Alberto Vassallo, Alessandra Modi, Andrea Quagliariello, Giovanni Bacci, Teresa Faddetta, Michele Gallo, Aldesia Provenzano, Andrea La Barbera, Giovanna Lombardo, Valentina Maggini, Fabio Firenzuoli, Marco Zaccaroni, Giuseppe Gallo, David Caramelli, Carla Aleo Nero, Franco Baldi, Renato Fani, Antonio Palumbo Piccionello, Sandra Pucciarelli, Anna Maria Puglia, Luca Sineo

**Affiliations:** a School of Biosciences and Veterinary Medicine, University of Camerino, Camerino (MC), Italy; b Department of Biology, University of Florence, Florence (FI), Italy; c Department of Comparative Biomedicine and Food Science, University of Padova, Legnaro (PD), Italy; d Department of Biological, Chemical and Pharmaceutical Sciences and Technologies, University of Palermo, Palermo (PA), Italy; e Department of Molecular Sciences and Nanosystems, Ca’ Foscari University of Venice, Venezia Mestre (VE), Italy; f Department of Clinical and Experimental Biomedical Sciences “Mario Serio,” University of Florence, Florence (FI), Italy; g Unit of Medical Genetics, IRCCS Ospedale Policlinico San Martino, Genoa (GE), Italy; h Research and Innovation Center in Phytotherapy and Integrated Medicine, Tuscany Region, Careggi University Hospital, Florence (FI), Italy; i Soprintendenza ai Beni culturali e ambientali di Palermo, Palermo (PA), Italy; Tianjin University

**Keywords:** paleomicrobiology, bacterial spores, aureothin, *Streptomyces*, *Nocardioides*, Palermo

## Abstract

The urban plan of Palermo (Sicily, Italy) has evolved throughout Punic, Roman, Byzantine, Arab, and Norman ages until it stabilized within the borders that correspond to the current historic center. During the 2012 to 2013 excavation campaign, new remains of the Arab settlement, directly implanted above the structures of the Roman age, were found. The materials investigated in this study derived from the so-called Survey No 3, which consists of a rock cavity of subcylindrical shape covered with calcarenite blocks: it was probably used to dispose of garbage during the Arabic age and its content, derived from daily activities, included grape seeds, scales and bones of fish, small animal bones, and charcoals. Radiocarbon dating confirmed the medieval origin of this site. The composition of the bacterial community was characterized through a culture-dependent and a culture-independent approach. Culturable bacteria were isolated under aerobic and anaerobic conditions and the total bacterial community was characterized through metagenomic sequencing. Bacterial isolates were tested for the production of compounds with antibiotic activity: a *Streptomyces* strain, whose genome was sequenced, was of particular interest because of its inhibitory activity, which was due to the Type I polyketide aureothin. Moreover, all strains were tested for the production of secreted proteases, with those belonging to the genus *Nocardioides* having the most active enzymes. Finally, protocols commonly used for ancient DNA studies were applied to evaluate the antiquity of isolated bacterial strains. Altogether these results show how paleomicrobiology might represent an innovative and unexplored source of novel biodiversity and new biotechnological tools.

**IMPORTANCE** One of the goals of paleomicrobiology is the characterization of the microbial community present in archaeological sites. These analyses can usually provide valuable information about past events, such as occurrence of human and animal infectious diseases, ancient human activities, and environmental changes. However, in this work, investigations about the composition of the bacterial community of an ancient soil sample (harvested in Palermo, Italy) were carried out aiming to screen ancient culturable strains with biotechnological potential, such as the ability to produce bioactive molecules and secreted hydrolytic enzymes. Besides showing the biotechnological relevance of paleomicrobiology, this work reports a case of germination of putatively ancient bacterial spores recovered from soil rather than extreme environments. Moreover, in the case of spore-forming species, these results raise questions about the accuracy of techniques usually applied to estimate antiquity of DNA, as they could lead to its underestimation.

## INTRODUCTION

The urban plan of Palermo (*Panormus* as a Phoenician-Punic city) has evolved throughout Punic, Roman, Byzantine, Arab, and Norman ages until it stabilized within the borders that today correspond to the current historic center. Generally speaking, the centrality of this area from the 2nd century BCE to the 3rd century CE was well known so far (1). The 2012 to 2013 excavations (by Aleo Nero of the BBCCAA Superintendency, Archaeological Section of Palermo), carried out on the occasion of urban works, have brought out a new building phase starting from the Arab age that was not highlighted before and that is implanted directly above the structures of the Roman age. Wall structures and abundant Arabic ceramics of different types dating from the mid-10th to the mid-11th century CE have been discovered.

**FIG 1:**
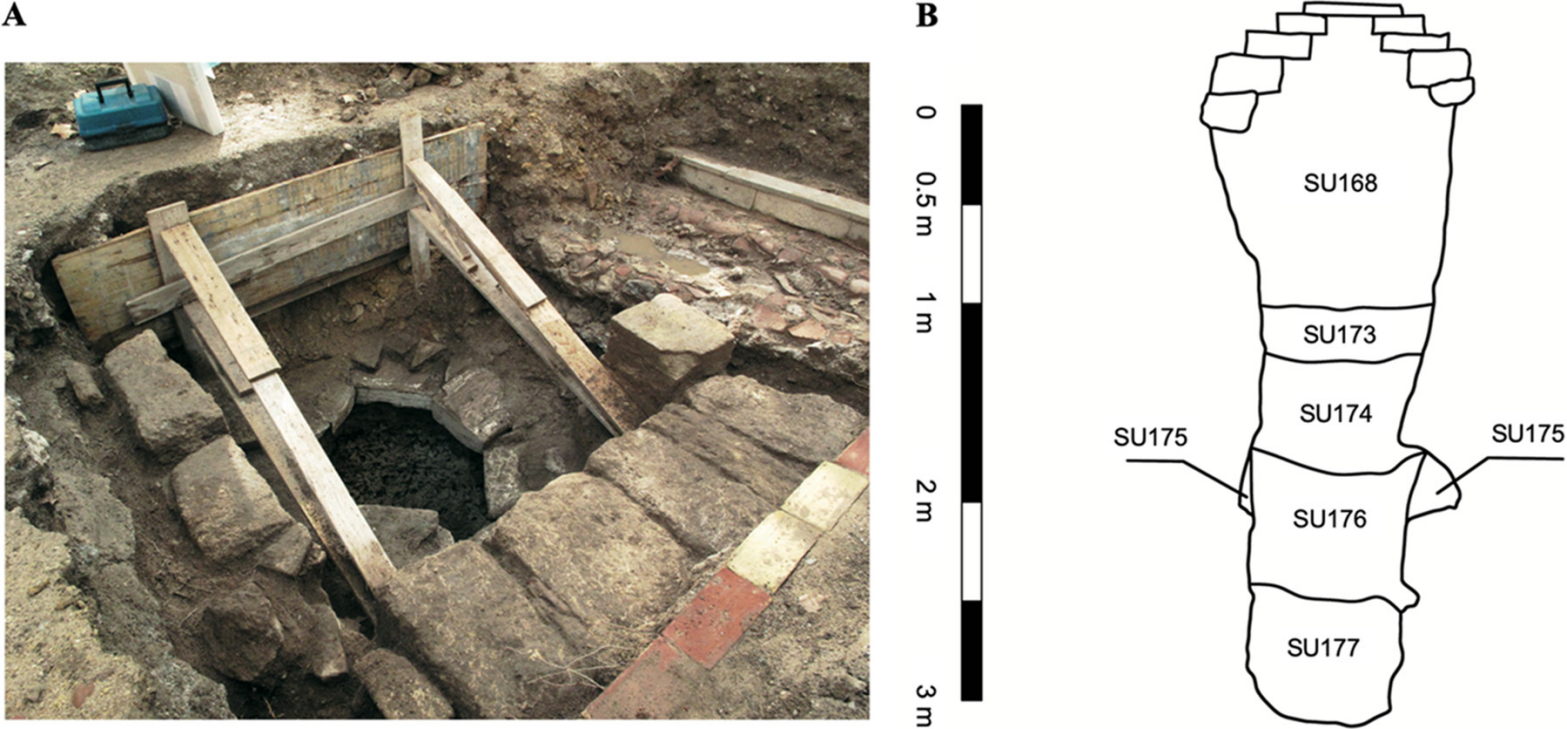
Survey No 3 site. A: Top view picture. B: Vertical stratigraphic section (2). Six stratigraphic units were identified and described as follows (from up to bottom): SU168 had inconsistent materials attributable to infiltration activities occurred during modern age; SU173 had a loose consistency and included loose materials; SU174 had a loose consistency and contained tiles, a few ceramic fragments, and fragments of cocciopesto; SU175 had a very compact consistency, a greenish color, and contained several ceramic fragments (one of these was relative to an Islamic overpainted amphora); SU176 (the stratigraphic unit investigated in this work) was black colored (burnt), with a relatively compact consistency (but slightly less than SU175), and included grape seeds, bone fragments, fish scales and bones, and coals; SU177 was very compact, beige colored, with rock fragments relating to the construction of the well.

The materials investigated in this study come from the so-called Survey No 3 (2), in particular from the stratigraphic unit 176 (hereinafter indicated as SU176) (Fig. 1). It consists in a rock cavity of subcylindrical shape (3.20 m deep and an average width of 0.80 m) covered with calcarenite blocks. This structure was used to dispose of garbage during the Arabic age, as evidenced by a layer of thousands of perfectly preserved seeds (particularly grape seeds), together with other organic remains such as scales and bones of fish, small animal bones, and charcoals. Thus, this deposit was used to get rid of daily activity remains. The soil is finely grainy and highly organic. SU176 is sealed by three distinct successive stratigraphic units (Fig. 1B).

The layers of soil and debris have very informative potential and are very useful for the possibility of reconstructing the ancient environment and landscape. Moreover, they offer the opportunity to carry out wide-spectrum analyses, including biological ones. In this context, paleomicrobiology investigations can often provide valuable and complementary information about past events, taking advantage of next generation sequencing-based approaches (3, 4). Although most examples of this multidisciplinary approach regard the study of the natural history of human infectious diseases and pathogens to understand their evolution and virulence (5–8), other investigations have been conducted to provide evidences about past human activities (9, 10), to date environmental changes (11) and conduct ecological studies about past ecosystems (12, 13), to detect traces of bacterial infections in ancient animal remains (14), and to reconstruct events occurred in past human settlements (15).

Soil is recognized as one of the richest sources of bacteria producing bioactive compounds (16). In this regard, recent examples of the successful isolation from soil of bacteria producing interesting and promising biomolecules (e.g., compounds with antibacterial, antifungal, antiviral, anti-inflammatory, and anticancer activities) are available in literature (17–22). For instance, such bacteria have been shown to inhibit growth of human and animal pathogenic microorganisms (23–26). Notably, most of the isolated producer bacteria belong to the phylum *Actinomycetota*, in particular to the genus *Streptomyces* (27, 28). Thus, the aims of this work were to characterize the composition of the bacterial community of the samples collected from Survey No 3 by using both culture-dependent and culture-independent approaches, and to screen culturable bacteria for their ability to produce bioactive molecules and hydrolases with biotechnological potential.

## RESULTS

### Analysis of SU176 content.

Content analysis and dating indicated that during the High Middle-Ages, the pit described in this work might have been used to dispose rubbish. Indeed, besides soil, samples contained 210 grape seeds, some metal wastes, a fragment of upper bivalve valves (possibly derived from the biogenic limestone), and some animal bone fragments (i.e., three sesamoid bones of Canis familiaris, bones, teeth and scales of fish, and a fragment of sheep long bone). In the case of the grape seeds, such a quantitatively large finding is very rare. The medieval origin of this site was confirmed by AMS ^14^C dating: in fact, calibrated radiocarbon dating of the grape seeds gave results reported in Table 1.

**TABLE 1:** Radiocarbon dating regarding grape seeds found in SU176

Radiocarbon age	908 ± 33 BP (before Present)
δ^13^C	−34 ± 2‰
1 Sigma	CE 1044 – CE 1099
2 Sigma	CE 1034 – CE 1193

The morphometric analysis of the grape seeds identified a low level of variability inside the sample and an intermediate position in terms of fine morphology between current wild *Vitis* and Sicilian cultivars. This could indicate a rather uniform provenience of the Vitis vinifera seeds (G. Lombardo personal communication) and presumably the short duration, in terms of use, of this stratum.

### Isolation and identification of bacteria from sample of SU176.

Bacteria were isolated under aerobic and anaerobic conditions: the total viable count of aerobic bacteria in the soil sample was 2.088 × 10^4^ CFU/g, while that of anaerobic ones was 5 × 10^7^ CFU/g. Isolated colonies were clustered by phenotype and two representatives of each were used for further analyses. Thus, 23 isolates were considered. Sequencing and analysis of genes encoding the 16S rRNA (16S rDNAs) revealed that bacterial isolates were affiliated with the following genera: *Arthrobacter*, *Bacillus*, *Nocardia*, *Nocardioides*, *Paenibacillus*, and *Streptomyces* (Table 2, Table S1).

**TABLE 2:** Strains isolated from SU176. + and – indicate isolation in aerobic and anaerobic conditions, respectively

Isolate	Accession no.	Aerobiosis	Isolation medium	Genus
*Arthrobacter* sp. AV21P	MW282143	+	SFM	*Arthrobacter*
*Bacillus* sp. AV1	MW282129	+	LB agar	*Bacillus*
*Bacillus* sp. AV11	MW282135	+	LB agar	
*Bacillus* sp. AV13	MW282136	+	LB agar	
*Bacillus* sp. AV15	MW282137	+	LB agar	
*Bacillus* sp. AV16	MW282138	+	LB agar	
*Bacillus* sp. AVA	MW282147	−	FeRid	
*Bacillus* sp. AVF	MW282151	−	Postgate C	
*Nocardia* sp. AV9	MW282133	+	LB agar	*Nocardia*
*Nocardia* sp. AV10	MW282134	+	LB agar	
*Nocardioides* sp. AV21	MW282142	+	SFM	*Nocardioides*
*Nocardioides* sp. AV22	MW282144	+	SFM	
*Nocardioides* sp. AV23	MW282145	+	SFM	
*Nocardioides* sp. AV24	MW282146	+	SFM	
*Paenibacillus* sp. AVB	MW282148	−	FeRid	*Paenibacillus*
*Paenibacillus* sp. AVC	MW282149	−	FeRid	
*Paenibacillus* sp. AVD	MW282150	−	FeRid	
*Streptomyces* sp. AV2	MW282130	+	LB agar	*Streptomyces*
*Streptomyces* sp. AV6	MW282131	+	LB agar	
*Streptomyces* sp. AV8	MW282132	+	LB agar	
*Streptomyces* sp. AV19	MW282139	+	SFM	
*Streptomyces* sp. AV20A	MW282140	+	SFM	
*Streptomyces* sp. AV20B	MW282141	+	SFM	

### Metagenomic analysis and analysis of ancient DNA.

Shotgun sequencing of metagenome was performed to characterize the bacterial community of the soil sample and to investigate the hypothesis that isolated bacteria were ancient. Microorganisms are normally heterogeneously distributed inside microaggregates and macroporosities of the soil (29) and, under specific physical-chemical conditions, soil particles can bind DNA fragments of diverse lengths, enabling the preservation of ancient DNA (aDNA) molecules over time (30–32). However, DNA extraction and purification from sediments is hindered by the presence of mineral and organic components of the soil matrix and by the numerous inhibitory compounds commonly found in environmental samples (33). Thus, two different extraction protocols optimized for the recovery of ancient DNA (i.e., method PS and method D, as described in Materials and Methods) were used. The highest DNA yield was obtained through method D, with a concentration of 2.44 ng/μL, while extraction with method PS yielded a DNA concentration of 1.49 ng/μL (Table S2).

**FIG 2:**
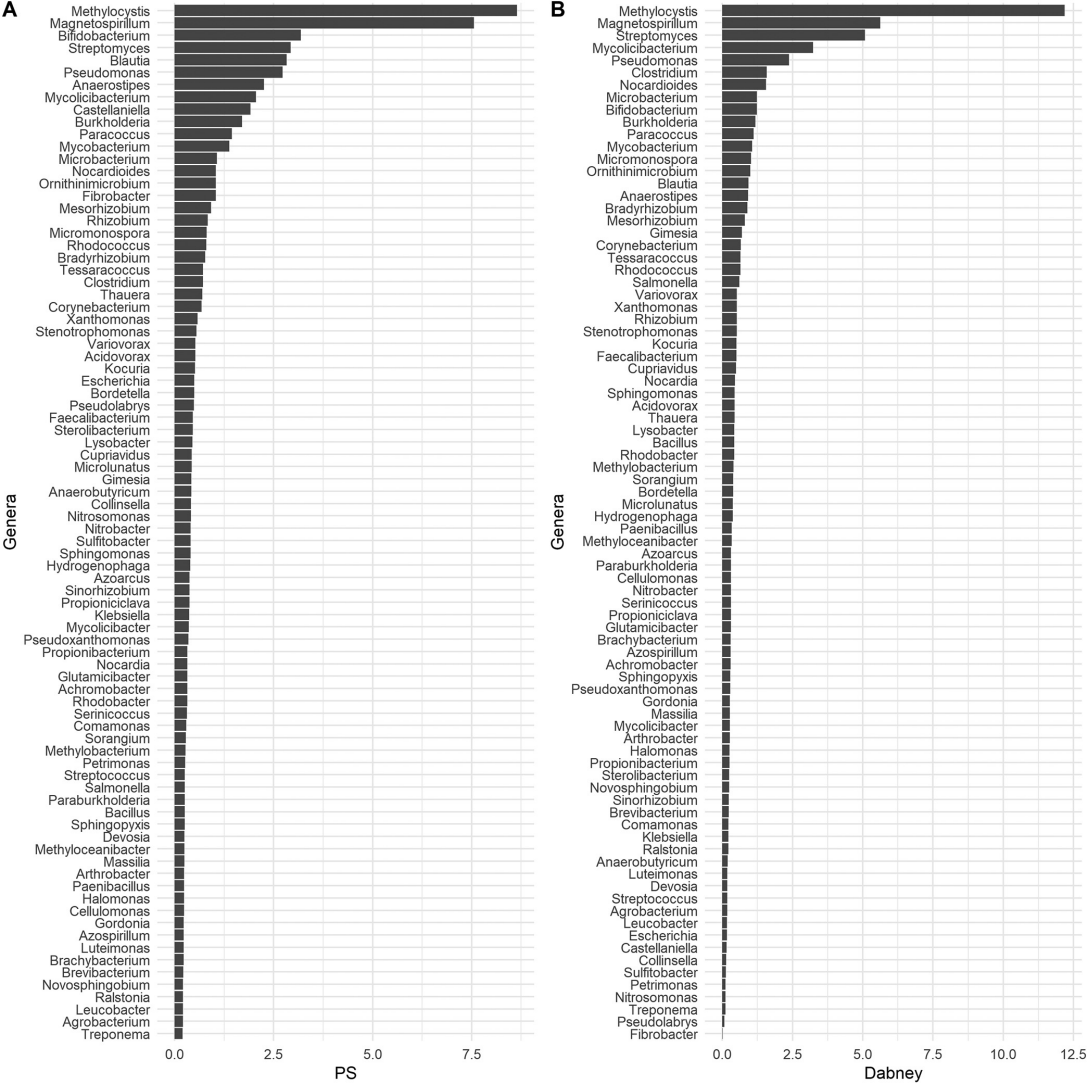
Bacterial genera distribution. The histograms represent the relative abundance of the most abundant genera in the samples extracted with the two protocols reported in Materials and Methods (Panel A: method PS; Panel B: method D). Genera with a relative abundance higher than 0.2% in each sample are reported in the plot.

After shotgun sequencing, 20,308,706 raw reads were obtained (10,487,426 for extraction D and 8,655,023 for PS). Microbial taxonomic profiling showed that the two extraction methods produced similar results regarding the bacterial taxonomic composition (Table S3). Bacterial genera having a relative abundance >0.2% are represented in Fig. 2, as this threshold was chosen for its capacity to describe >50% of the overall biodiversity of each sample. The bacterial community identified with metagenomic analysis included also all the genera that the isolated bacteria belonged to (i.e., *Arthrobacter*, *Bacillus*, *Nocardia*, *Nocardioides*, *Paenibacillus*, and *Streptomyces*; Table 2). Moreover, it is important to notice that, although their DNA was detected with this analysis, bacteria belonging to the genera that are commonly present in soil samples (e.g., *Agrobacterium*, Pseudomonas, *Stenotrophomonas*, etc.) and that are generally cultivable in nonselective media (like those used in this work) were not isolated, probably because they could be unviable or in a viable but not-culturable (VBNC) state (34, 35). Thus, these findings supported the hypothesis that isolated bacteria were ancient.

To this aim, antiquity of DNA was then investigated through the analysis of deamination patterns. Data showed that DNA of only eight species could be considered ancient (Table S4): Anaerostipes hadrus, Bifidobacterium angulatum, *Blautia* sp. SC05B48, Castellaniella defragrans, Clostridium perfringens, Escherichia coli, Methylocystis rosea, and *Methylocystis* sp. SC2. Except for *C. perfrigens* (36), none of these species are able to produce spores (37–44): in the case of *C. perfrigens* it cannot *a priori* be excluded that, besides its DNA, even its spores were present in the soil sample and that they would have been able to germinate if seeded in the appropriate conditions (e.g., optimal medium and anaerobiosis).

### Antimicrobial activity and identification of bioactive compounds.

**FIG 3:**
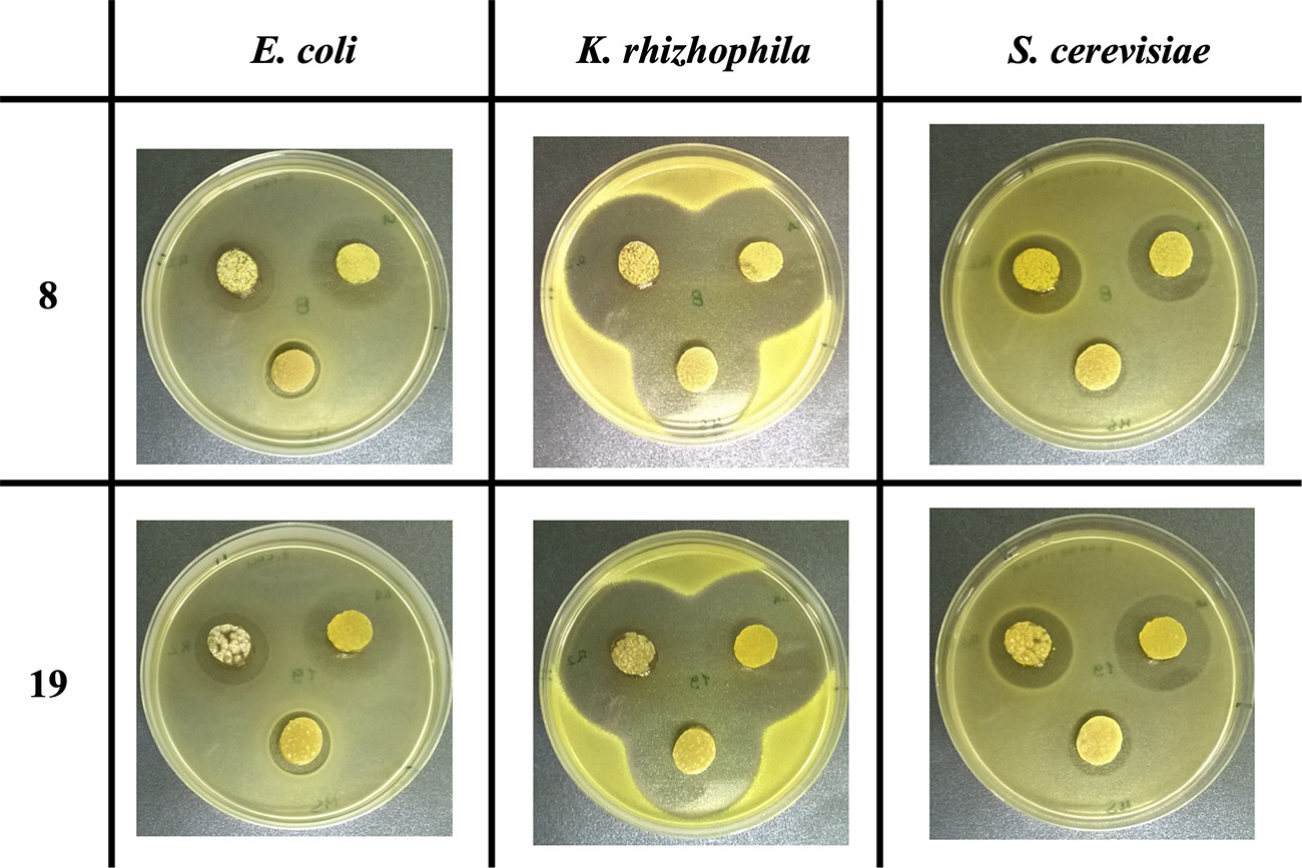
Antimicrobial activity assay using the agar plug method. *Streptomyces* sp. AV8 and *Streptomyces* sp. AV19 were cultivated on R2YE (left upper plug), LB Agar (right upper plug) and SFM (lower plug).

The production of bioactive compounds by the 23 isolates was assayed against Escherichia coli, Kocuria rhizophila, and Saccharomyces cerevisiae that were chosen because they are a Gram-negative bacterium, a Gram-positive bacterium, and a eukaryote, respectively. Isolates were cultivated on three different media to evaluate the possible influence of nutrients on antimicrobial synthesis. Among them, only those belonging to the genus *Streptomyces* exerted an antimicrobial activity. In particular, *Streptomyces* sp. AV8 and *Streptomyces* sp. AV19 were of particular interest because they produced the largest inhibition halos (Fig. 3, Table S5).

As shown in Fig. 3, both strains exerted a strong growth inhibition of the Gram-positive *K. rhizophila* and, although to a lesser extent, even the growth of E. coli and S. cerevisiae was inhibited. Notably, their inhibition halos had also almost identical diameters (Table S5), and alignment of their 16S rDNA partial sequences (100% identity) suggested that these two isolates belonged to the same species and/or strain.

**FIG 4:**
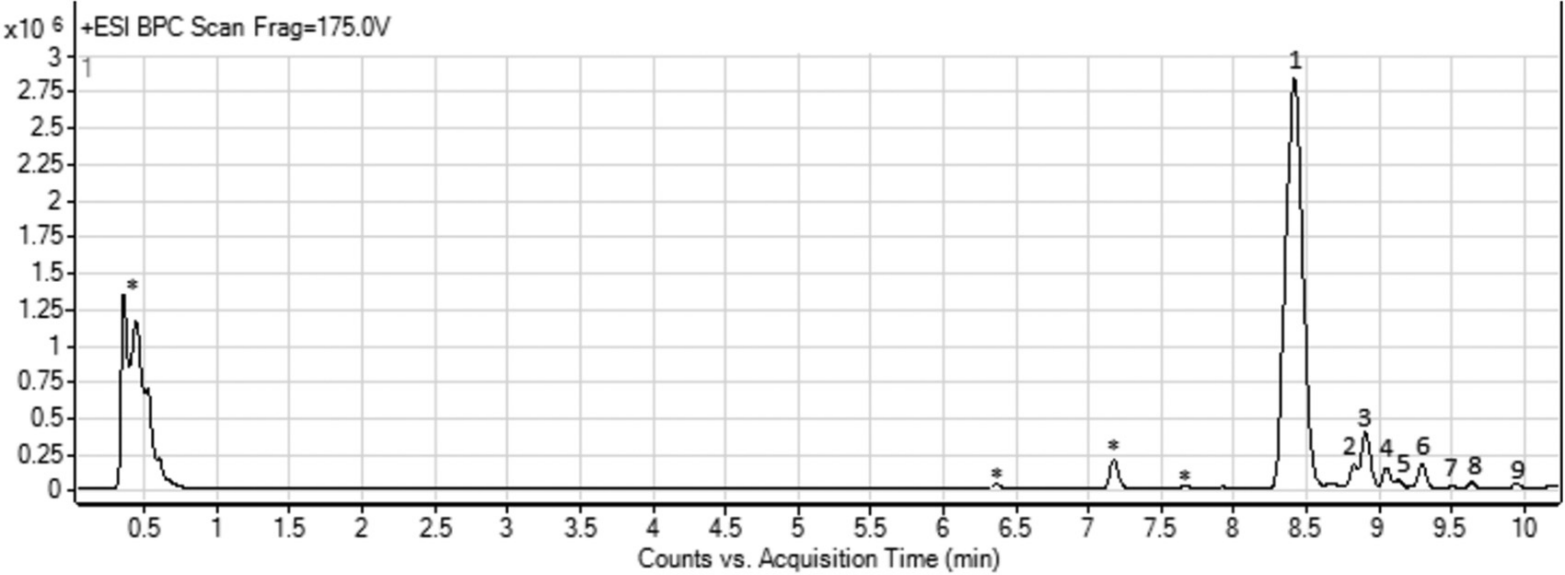
Representative HPLC/MS trace for methanolic extract of *Streptomyces* sp. AV19. *, peaks referred to the culture medium.

Hence, on the basis of these results, a screening of bioactive compounds produced by *Streptomyces* sp. AV19 grown on R2YE was performed, and methanolic extracts whose antimicrobial activity was verified by microbiological assays against *K. rhizophila* (Fig. S1) were analyzed by means of HPLC/ESI/Q-TOF (High Performance Liquid Chromatography/Electron Spray Ionization/Quadrupole-Time of Flight). In Fig. 4, the HPLC trace for *Streptomyces* sp. AV19 is reported.

**FIG 5:**
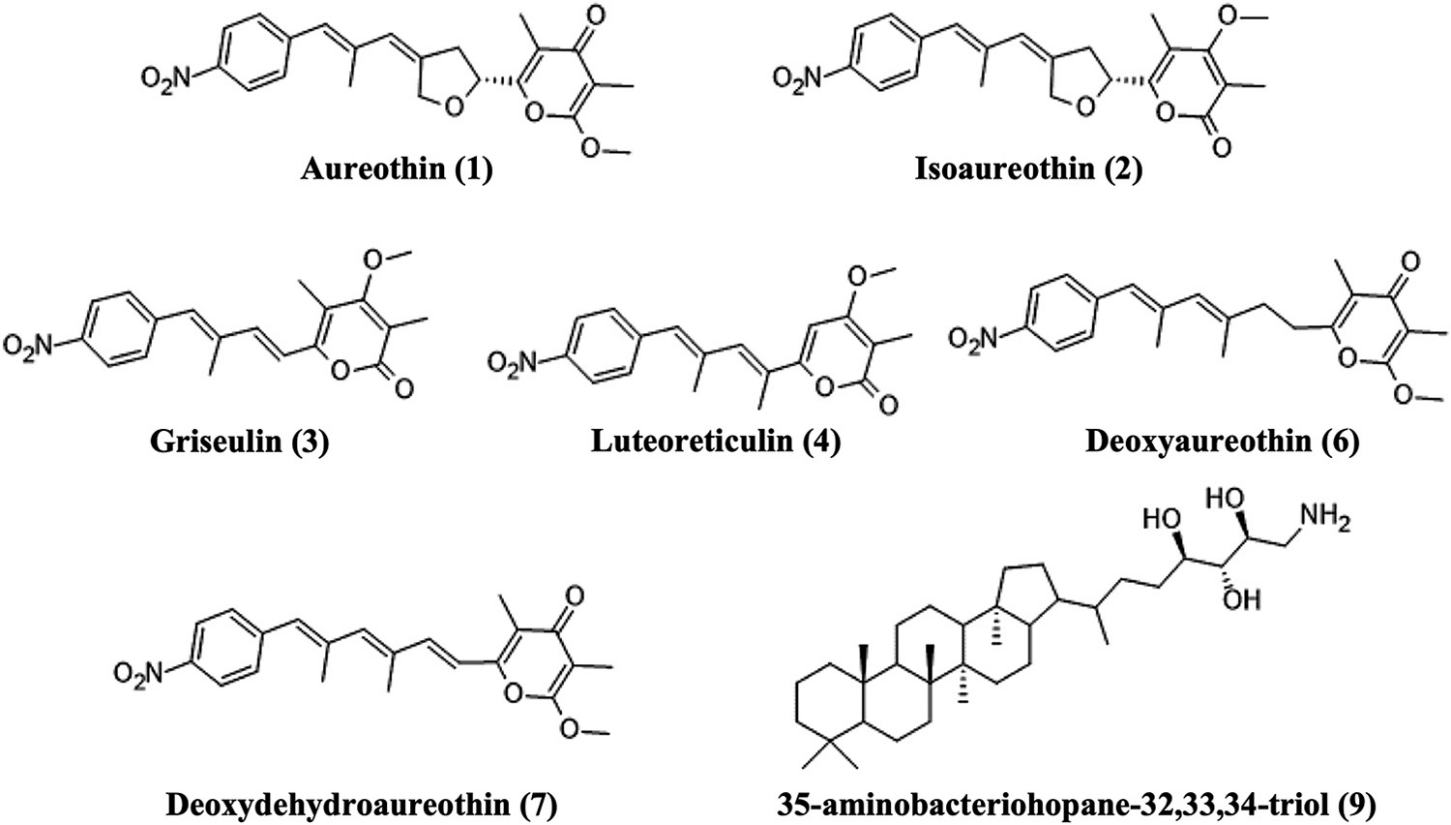
Chemical structure of aureothin and related metabolites. Numbers refer to peaks depicted in Fig. 4.

The main compound found in the extract was the Type I polyketide antibiotic aureothin (peak 1 in Fig. 4) accompanied by some compounds related to its production (peaks 2 to 8 in Fig. 4) and a membrane hopanoid (peak 9 in Fig. 4) (45–48) (Fig. 5, Table 3, Fig. S2).

**TABLE 3:** Metabolites identified in the methanolic extract of *Streptomyces* sp. AV19 culture through HPLC/MS. Peaks are numbered in agreement with the HPLC trace depicted in Fig. 4

Peak	Compound	Rt (min)	Calcd. mass	Exp. mass	Formula
1	Aureothin	8.37	398.1598 [M + H]^+^	398.1596	C_22_H_23_NO_6_
2	Isoaureothin	8.82	398.1598 [M + H]^+^	398.1602	C_22_H_23_NO_6_
3	Griseulin^*a*^	8.87	342.1336 [M + H]^+^	342.1334	C_19_H_19_NO_5_
4	Luteoreticulin^*a*^	9.05	342.1336 [M + H]^+^	342.1330	C_19_H_19_NO_5_
5	Aureothin isomer	9.14	398.1598 [M + H]^+^	398.1596	C_22_H_23_NO_6_
6	Deoxyaureothin	9.30	384.1805 [M + H]^+^	384.1805	C_22_H_25_NO_5_
7	Deoxydehydroaureothin	9.50	382.1649 [M + H]^+^	382.1646	C_22_H_23_NO_5_
8	Deoxydehydroaureothin isomer	9.64	382.1649 [M + H]^+^	382.1650	C_22_H_23_NO_5_
9	35-Aminobacteriohopane−32,33,34-triol	10.52	546.4881 [M + H]^+^	546.4877	C_35_H_63_NO_3_

a Tentative identification.

### Sequencing of *Streptomyces* sp. AV19 genome.

Genomic DNA of *Streptomyces* sp. AV19 was extracted and sequenced, obtaining a draft genome with 8 contigs and N50 = 4,053,991 bp: the total length was 7,671,946 bp and a GC content 72.15%, thus both parameters being consistent with other *Streptomyces* genomes. The phylogenetic analysis based on the complete 16S rDNA of *Streptomyces* sp. AV19 showed that the closest species is Streptomyces luteireticuli (Fig. S3). The antiSMASH program (version 6.1.1) (49) was used to identify secondary metabolite biosynthetic gene clusters, selecting ‘strict’ as detection strictness. Data obtained revealed the presence of 36 clusters in *Streptomyces* sp. AV19 genome (Table S6). The most represented gene cluster type was Polyketide Synthase (PKS), followed by Non-Ribosomal Peptide Synthetase (NRPS). In agreement with the HPLC/MS data, aureothin biosynthetic gene cluster was detected, having a 100% similarity with the Streptomyces thioluteus one (50–52). Moreover, in the case of coelichelin biosynthetic gene cluster, a 100% similarity was found with the Streptomyces coelicolor one (53–55).

### Protease activity.

**FIG 6:**
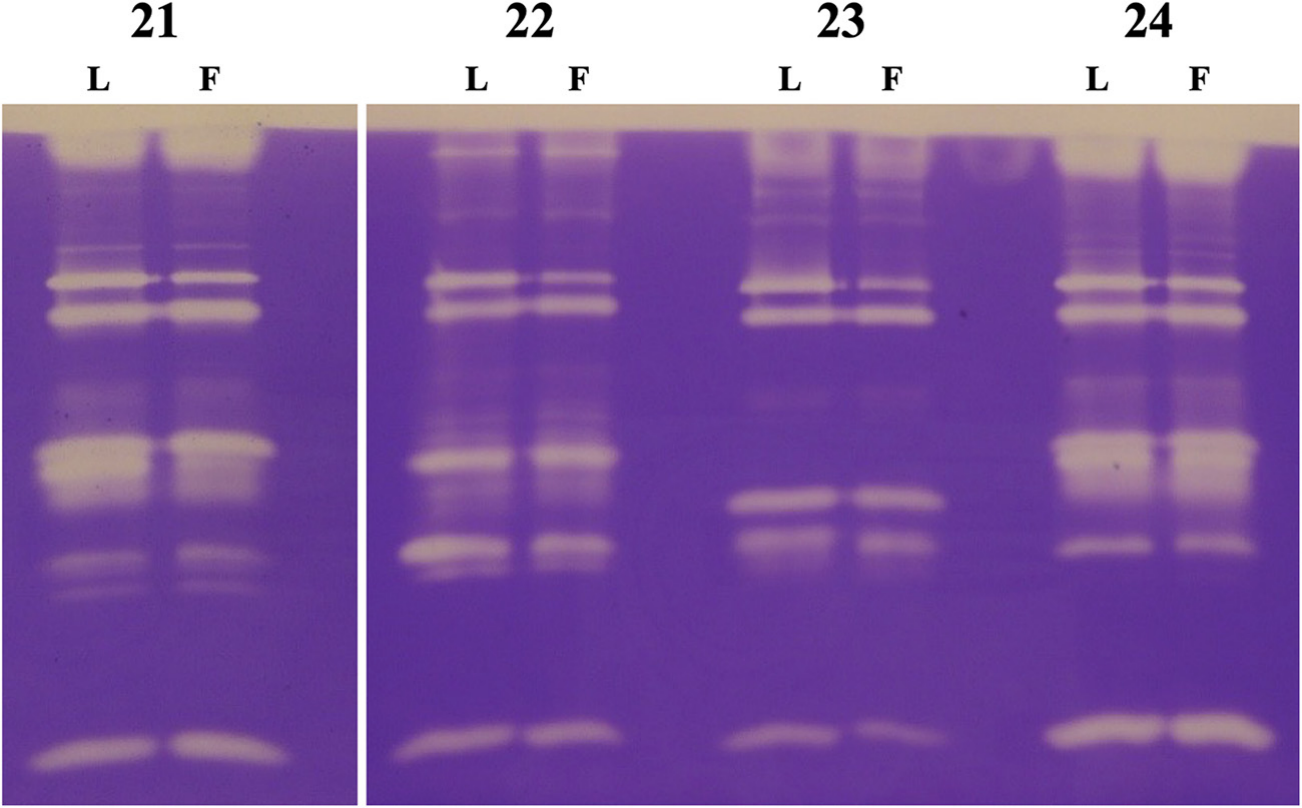
Extracellular proteases secreted by bacterial isolates belonging to the genus *Nocaridioides* (i.e., *Nocaridioides* sp. AV21, *Nocaridioides* sp. AV22, *Nocaridioides* sp. AV23, and *Nocaridioides* sp. AV24). L and F indicate the medium used, LB and FermII, respectively.

All isolates were tested for the production of secreted proteases through a gel zymography exploiting gelatin as the substrate. Although this assay showed that most of the isolates produced this kind of hydrolases, those belonging to the *Nocardioides* genus (i.e., *Nocardioides* sp. AV21, *Nocardioides* sp. AV22, *Nocardioides* sp. AV23, and *Nocardioides* sp. AV24) had the most complex patterns (Fig. 6). As shown in Fig. 6, for each isolate no differences were observed between the two media used for cultivation. Noteworthy, the four couples of patterns were different one another, suggesting that the four isolates belonged to different species and/or strains. Moreover, since spent media were tested without further processing (e.g., concentration), the intensity of the bands suggested an interesting potential of these bacteria for future biotechnological applications.

## DISCUSSION

In this work the characterization of the bacterial community of an ancient soil retrieved from an Arabic site located in *Piazza della Vittoria* in Palermo (Sicily, Italy) has been described. The isolation of culturable bacteria was performed in both aerobic and anaerobic conditions, yielding to the growth of strains belonging to *Arthrobacter*, *Bacillus*, *Nocardia*, *Nocardioides*, *Paenibacillus*, and *Streptomyces* genera. They belong to either *Actinomycetota* or *Bacillota* phyla (56) and are commonly recognized as soil-dwelling bacteria. Notably, bacteria belonging to all these genera include species able to produce spores (i.e., *Bacillus*, *Nocardia*, *Nocardioides*, *Paenibacillus*, and *Streptomyces*) (28, 57) or dormant forms (i.e., *Arthrobacter*) (58). This peculiarity might be considered a strong suggestion that all described procedures, from sampling to isolation, were performed avoiding any external contaminations and, above all, that the sampled site was really isolated from the surrounding environment, at least in more recent times. Indeed, neither common environmental and laboratorial bacteria (e.g., E. coli, Staphylococcus, *Agrobacterium*, *Burkholderia*, Pseudomonas, *Rhizobium*, etc.) nor fungi, for example molds, were isolated, although normally able to grow in the nonselective media used in this work. However, in the case of fungi, it should be considered that specific and/or selective media for their isolation were not used here and that the adopted cultural conditions might have not supported their growth.

Metagenomic analysis of the soil sample revealed the existence of a more complex bacterial composition. It is quite possible that bacteria detected by metagenomic analysis, but not isolated, (i) were unviable and only their DNA endured in the soil, or (ii) they could be in a VBNC state, or (iii) they were viable but they did not grow due to the adopted culturing conditions or to inhibition activity by other growing bacteria. Nevertheless, it is not possible to know whether the site has remained constantly isolated along the centuries. In this regard, it cannot *a priori* be excluded that the site could have been contaminated because of disparate and unpredictable events (e.g., water infiltration, accidental opening, colonization by arthropods normally inhabiting soil, etc.) after it stopped being used for its original purpose and it was abandoned and eventually forgotten.

Isolation of bacteria potentially able to produce spores or dormant forms might suggest that these isolates derived by germination of ancient spores. Shotgun sequencing and degradation patterns analysis identified a group of eight species with the expected features of DNA isolated from ancient organisms (i.e., short fragments and high frequency of misincorporation at the ends of the molecules [59]), with none of them being related to our isolated bacteria. Thus, antiquity of our isolates would be fairly questionable. However, it should be considered that the antiquity of DNA is normally qualitatively evaluated on the basis of the degradation that occurs over time under specific environmental and taphonomic conditions (60–63). Moreover, degradation kinetics process is well-known in ancient bones and teeth (64–66) rather than in other biological substrates. Indeed, the absence of damage patterns could be potentially explained by the structure of spores: DNA packed within spores is probably less (or not at all) exposed to environment. Thus, it might be possible that evaluation of DNA deamination patterns is not the most reliable method to determine antiquity of DNA derived by organisms producing resistant forms such as spores, as this approach could possibly lead to its underestimation. Albeit these considerations are probably reasonable, data reported in this work do not allow to undoubtedly confirm that bacteria described here were truly ancient.

Although works regarding the successful, and in some cases controversial, germination of very ancient *Bacillus* spores have been reported, environmental conditions described thereof do not resemble ours (67–72). Indeed, our isolates derived from a buried, although isolated, soil sample rather than uncommon and extreme environments, such as extremely old inclusions in amber or permafrost. If confirmed, this might represent an example of germination of ancient spores recovered from a soil sample and produced by bacteria belonging to other genera than *Bacillus*, whose longevity and resistance against abiotic stresses are extensively reported and under investigation, even with a 500 year-long experiment (73–75). Noteworthy, in case of confirmation, this would be, to the best of our knowledge, the first example of germination of ancient spores produced by the actinomycete *Nocardia*, *Nocardioides*, and *Streptomyces* strains.

Among the isolates, only streptomycetes exhibited the production of bioactive compounds with antimicrobial activity. In detail, *Streptomyces* sp. AV19 was of particular interest because of its strong inhibitory effect on the growth of target organisms. HPLC/MS procedures allowed to identify the Type I polyketide aureothin as the antibiotic compound produced by this strain. In agreement with experimental data, sequencing of its genome revealed the presence of the aureothin biosynthetic gene cluster sharing a 100% degree of similarity with that of *S. thioluteus*, the only known producer of this antibiotic until now. Moreover, the analysis of the *Streptomyces* sp. AV19 complete genome also revealed the presence of 38 predicted gene clusters for the biosynthesis of secondary metabolites, such as polyketides, nonribosomal peptides, and siderophores.

Finally, this work gave us the opportunity to test the presence of features amenable of further studies and which could be exploited for biotechnological purposes. This is the case of the sets of secreted proteases produced by *Nocardioides* isolates. Indeed, although commonly found in soil sample, this genus is still poorly characterized, especially from a molecular viewpoint, and, necessarily, new experiments will be performed to exploit its full potential. At this regard, next steps will take advantage of high-throughput procedures such as third-generation sequencing of DNA and phenotype microarray.

In conclusion, we underline the importance of having found daily wastes in a stratigraphically limited situation and sealed by depositions and later works. The experimental approach applied in this work is an example of how ancient bacterial strains might represent new sources of biochemical and metabolic capabilities potentially useful for modern biotechnology, demonstrating once again the need for and importance of a multidisciplinary approach to solve an archaeological problem.

## MATERIALS AND METHODS

### Sampling.

Two samples, consisting in 500 g of soil collected from the pit located in *Piazza della Vittoria* in Palermo, were examined. They were harvested using sterile scalpels and containers and derived from a defined and sealed stratum inside the pit. Samples were processed immediately upon arrival at the laboratory the same day of collection. The aliquot used for DNA extraction was kept in ice to avoid possible degradation and sent to a dedicated ancient DNA facility. The materials in the soil samples were analyzed under stereo microscope (Leica) and grape seeds coming from the stratigraphic unit underwent AMS ^14^C dating (Laboratory INNOVA Scarl. Caserta, Italy).

### Isolation of bacteria in aerobic conditions.

One g of sample was resuspended in 1 mL of sterile 0.9% wt/vol NaCl solution and 100 μL of 10^−1^ and 10^−2^ dilutions were plated on LB agar (Miller’s LB broth base, Invitrogen) and SFM (20 g/L mannitol, 20 g/L soy flour, 20 g/L Bacto agar) (76). CFU were enumerated upon incubation in standard atmosphere at 30°C for 3 to 7 days. Three independent replicates from the same soil sample were processed. Colonies were repeatedly streaked on fresh medium until pure cultures were obtained.

### Isolation of bacteria under anaerobic conditions.

Bacteria belonging to the functional group of Fe(III) reducing bacteria (FeRB) were isolated using an anaerobic medium (FeRid) containing: 1.0 g/L Fe citrate, 1.5 g/L peptone, 0.6 g/L NaH_2_PO_4_, 2.5 g/L NaHCO_3_, 1.5 g/L NH_4_Cl, 0.1 g/L CaCl_2_ · 2H_2_O, 0.1 g/L KCl, 0.1 g/L MgCl_2_ · 6H_2_O, 0.005 g/L MnCl_2_ · 6H_2_O, 0.001 g/L NaMoO_4_ · 2H_2_O, pH 7.0. The medium was sterilized and cooled down under nitrogen flux, then 100 mL of medium were distributed in 10 different sealed vials. The soil sample (100 mg) was suspended in 3 mL of sterile water, injected in the sealed vials and incubated at 28°C.

Bacteria belonging to the functional group of sulfate reducing bacteria (SRB) were isolated in the same way (at 28°C), but the previous medium was replaced by medium “Postgate C,” which contained: 0.5 g/L KH_2_PO_4_, 1.0 g/L NH_4_Cl, 4.5 g/L Na_2_SO_4_, 0.06 g/L CaCl_2_ · 6H_2_O, 0.06 g/L MgSO_4_ · 7H_2_O, 3.0 g/L Na lactate, 3.0 g/L Na acetate, 1.0 g/L yeast extract, 0.1 g/L FeSO_4_ · 7H_2_O, 0.3 g/L Na citrate · 2H_2_O, pH 7.5.

Both FeRB and SRB were isolated by injecting 1 mL of culture into new fresh medium for five times in a row, spending 3 months for isolating FeRB and SRB in liquid media. At the end, 100 μL of culture were streaked on the corresponding agar plates for the final isolation.

### 16S rDNA amplification and sequencing.

Genera of all isolated strains described in this work were determined through sequencing of 16S rDNA. PCRs were performed in a 25 μL volume containing 1× PCR Reaction Buffer (Invitrogen), 3.5 mM MgCl_2_, 200 μM each dNTPs (Invitrogen), 0.2 μM each universal primer (27F 5′-AGAGTTTGATCMTGGCTCAG-3′ and 1492R 5′-TACGGYTACCTTGTTACGACTT-3′) and 1 U of *Taq* DNA polymerase Recombinant (Invitrogen). One μL of single colony thermal lysate was used as the template DNA: briefly, a bacterial colony was resuspended in 25 μL of TE buffer (10 mM Tris-HCl, 1 mM EDTA, pH 8) and incubated at 100°C for 5 min; then the lysate was incubated in ice for 5 min and centrifuged (5 min at 11,000 × *g*); the resulting supernatant was eventually used as the template DNA. The thermal cycle was 94°C for 3 min, followed by 30 cycles of 94°C for 45 sec, 50°C for 1 min and 72°C for 90 sec, and finally 72°C for 10 min. PCR products were purified using the Purelink Quick Gel Extraction and PCR Purification Combo kit (Invitrogen) and sequenced by BMR Genomics (Padua, Italy) through Sanger sequencing.

In the case of *Streptomyces* sp. AV19, a 16S rDNA-based phylogenetic analysis was performed. The complete 16S rDNA was used as BLAST query, choosing the 16S rRNA sequences (Bacteria and Archaea) database, and limiting the search to the species recognized as whorl-forming *Streptomyces* (Table S7) (77). Sequence alignments were downloaded using the Multiple Sequence Alignment (MSA) Viewer tool and the phylogenetic tree was constructed by using the phangorn R library (v.2.10) (78). The maximum likelihood method was used to reconstruct the tree (79) with 100 bootstrap and stochastic rearrangements. Different nucleotide substitution models were tested before choosing the one with the lowest BIC value. The phylogenetic tree was visualized using the online tool iTOL (80).

### Analysis of metagenomic DNA.

All molecular laboratory work was conducted in the dedicated ancient DNA (aDNA) facilities at the Laboratory of Anthropology (University of Florence), following strict guidelines to prevent contaminations. Blank controls were processed in parallel with samples for monitoring of potential reagent contamination. Additionally, before proceeding with DNA extraction, the archaeological sample was exposed to UV light for 10 min to sterilize the external surface.

Two different silica-based extraction methods were used to increase the chances of recovering ancient microbial DNA from the sample. Here, these two methods are designated D and PS, respectively.

**(i) Extraction method D.** This method is based on a digestion and purification protocol commonly used to extract highly fragmented DNA molecules from mineralize tissues (81). While this protocol was developed for the recovery of aDNA from bones and teeth, it was successfully applied on different biological materials (82–84). Approximately 50 mg of soil were digested overnight at 37°C in 1 mL of Extraction Buffer (0.5 M EDTA, 0.25 mg/mL Proteinase K, and 0.05% vol/vol Tween 20). After pelleting, DNA was purified and concentrated using a High Pure Extender Assembly column from the Viral Nucleic Acid kit (Roche), combining the supernatant with 10 mL of binding buffer (5 M GuHCl, 40% vol/vol isopropanol) and 400 μL of 3 M Na acetate. After two washing steps with wash buffer (20 mM NaCl, 2 mM Tris-HCL in ethanol), DNA was eluted twice in a final volume of 60 μL of elution buffer.

**(ii) Extraction method PS.** A standard microbial extraction protocol for DNA recovery from soil was applied using the DNeasy PowerSoil Pro kit (Qiagen) and following manufacturer instructions with some modifications. In brief, approximately 200 mg of sediment were resuspended in a solution prepared with 400 μL of 0.5 M EDTA and 100 μL of 20 mg/mL Proteinase K. The sediment suspension was added to a PowerBead Tube containing 750 μL of Solution CD1 and rotated for 4 h at room temperature, followed by bead beating at 3,200 rpm for 10 min. After pelleting, the supernatant was split into two equal aliquots to reduce clogging issues encountered when loading extraction lysates onto a single column (83). Each aliquot was combined with 7.5 mL of binding buffer and centrifuged twice in a Roche High Pure Extender Assembly column. After two washing steps, DNA was eluted in two rounds of 30 μL of elution buffer for a total volume of 60 μL. After extraction, DNA yield was quantified using Qubit 4 Fluorometer with dsDNA High Sensitivity kit (Invitrogen).

**(iii) Preparation of DNA library and sequencing.** Twenty μL of each extract (i.e., D and PS) were prepared for shotgun metagenome sequencing. Double-stranded Illumina libraries were constructed following a custom double-indexing protocol (85) optimized for ancient samples. No uracil DNA glycosylase (UDG) treatment was performed to retain misincorporation patterns that can be used to authenticate aDNA sequences (86). After 15 cycles of indexing PCR, qualitative and quantitative analysis of the libraries was executed with Agilent TapeStation (D1000 kit). The two libraries were pooled in equimolar amounts and sequenced by Illumina MiSeq in paired-end mode (2 × 75 + 8 + 8 run parameters).

**(iv) Bioinformatic analysis.** Sequencing data were demultiplexed and sorted according to the individual sample barcodes using Illumina bcl2fastq conversion software. Raw reads were processed for their quality, the adapter sequences were removed, and paired-end reads collapsed using AdapterRemoval (v2) (87) software with the following option: −*minlength* 30 −*minquality* 25 −*trimns −trimqualities −collapse*. After sequences duplicate removal with Prinseq (88), Kraken2 (v.2.0.8-beta) (89) was used for taxonomic identification.

A custom database updated to December 2020 of bacterial, viral, archaeal, and mitochondrial genomes from the NCBI Reference Sequence (RefSeq) database (https://www.ncbi.nlm.nih.gov/refseq/) was created. To avoid spurious classification, we masked reference genomes for low-complexity regions with Dustmasker (https://www.ncbi.nlm.nih.gov/books/NBK569845/). The output of Kraken2 was analyzed through Bracken software to estimate species read abundances (90). In order to evaluate the authenticity of the most abundant identified species, each sample read was aligned to its respective reference genomes deposited in the NCBI RefSeq database using only that species reported as “reference” or “representative.” Burrows-Wheeler Alignment (BWA) program was used for this aim, using the *aln* algorithm with high stringency (*-n* 0.1) (91). Aligned sequences were then investigated for their deamination profile using PMDtools (−*threshold* 1 –*requiremapq* = 30) (https://github.com/pontussk/PMDtools). For each species aligned, bam files were converted to bed using bedtools (https://bedtools.readthedocs.io/en/latest/) and we estimated the edit distance (-tag NM) that was subsequently used to compute the edit distance algorithm (−Δ%) to further confirm sequence authenticity (92). The Bracken output was used to investigate species distribution among samples using R software and phyloseq package (93).

### Genome sequencing.

Genomic DNA of *Streptomyces* sp. AV19 was extracted as previously reported (76) and sequenced with both Illumina and Nanopore technologies. Illumina library was prepared through a strategy based on enzymatic fragmentation to produce dsDNA fragments followed by end-repair, A-tailing, adapter ligation and library amplification using the Kapa Hyperplus kit (Kapa Biosystems). The obtained DNA library was sequenced with single-end 300 cycle strategy by NextSeq550 using the NextSeq550 high output v2 kit (Illumina Inc.). Nanopore library was prepared as previously described (94), adopting a PCR-free approach and using the protocol provided by Oxford Nanopore Technologies (ONT) (version NBE_9065_v109_revY_14Aug2019). One μg of DNA was repaired and end-prepped using the NEBNext Companion Module for Oxford Nanopore Technologies Ligation Sequencing (New England Biolabs). Five hundred ng of end-prepped DNA were barcoded using Native Barcoding Expansion 13-24 (ONT) and NEB Blunt/TA Ligase Master Mix (New England Biolabs). Finally, adapters were ligated and the DNA library was enriched with >3 kb-long fragments using the Long Fragment Buffer included in the Ligation Sequencing kit (ONT). DNA library was immediately sequenced using an R9.4.1 Flow Cell (ONT), previously primed with Flow Cell Priming kit (ONT). Sequencing was performed with a MinION Mk1B (ONT) and the MinKNOW software (v.21.10.4) for 72 h. Basecalling was performed using Guppy (v.4.3.4).

*De novo* hybrid assembly of the genome sequence was accomplished using Unicycler software (v. 0.4.8.0) in ‘conservative’ bridging mode (95), within a Galaxy environment. The draft genome has 8 contigs, a total length of 7,671,946 bp and N50 of 4,053,991 bp.

### Antibiotic activity assay.

All bacterial isolates were tested to assess the production of bioactive metabolites. The agar plug method was applied and Escherichia coli DH10B, Kocuria rhizophila ATCC 9341 and Saccharomyces cerevisiae DBY746 were used as target organisms. Briefly, isolates were cultivated on LB Agar, SFM, and R2YE (76) at 30°C for 24 to 48 h to obtain a homogeneous growth. Fresh cultures of target organisms were used to prepare suspensions in 0.9% wt/vol NaCl solution having OD_600_ = 1. Then, 180 μL of suspension were inoculated in 10 mL of LB Soft Agar (10 g/L NaCl, 5 g/L yeast extract, 10 g/L tryptone, 7 g/L Bacto agar) in case of E. coli and *K. rhizophila*, and in 10 mL of YPD Soft Agar (10 g/L yeast extract, 20 g/L peptone, 20 g/L glucose, 7 g/L Bacto agar) in case of S. cerevisiae (both media were previously melted and cooled before addition of microorganisms), and poured in Petri dishes. Then, a circular plug from the aforementioned cultures of testers was placed on the surface of solidified target organism cultures. E. coli and *K. rhizophila* plates were incubated at 37°C for 24 h, while those with S. cerevisiae were incubated at 30°C for 24 h. The production of antibiotics was qualitatively evaluated as the presence of growth inhibition halos surrounding the agar plugs. This assay was performed in triplicate and agar plugs of the corresponding solid media (i.e., without bacteria) were used as control.

### Reversed phase HPLC/ESI/Q-TOF HRMS (high resolution mass spectrometry) experiments.

Samples for HPLC/MS analysis were prepared by treating cultures of *Streptomyces* sp. AV19 grown on R2YE with MeOH (40 mL), under sonication for 30 min. The methanolic extract was centrifuged (4,000 × *g*, 10 min) and the supernatant was directly injected or diluted with mobile phase. Water and acetonitrile were of HPLC/MS grade. Formic acid was of analytical quality. The HPLC system was an Agilent 1260 Infinity. A reversed-phase C18 column (ZORBAX Extend-C18 2.1 × 50 mm, 1.8 μm) with a Phenomenex C18 security guard column (4 mm x 3 mm) was used. The flow rate was 0.4 mL/min and the column temperature was set to 30°C. The eluents were formic acid–water (0.1:99.9, vol/vol) (phase A) and formic acid–acetonitrile (0.1:99.9, vol/vol) (phase B). The following gradient was employed: 0 to 10 min, linear gradient from 5% to 95% B; 10 to 15 min, washing and reconditioning of the column to 5% B. Injection volume was 10 μL. The eluate was monitored through MS TIC. Mass spectra were obtained on an Agilent 6540 UHD accurate-mass Q-TOF spectrometer equipped with a Dual AJS ESI source working in positive mode. N_2_ was employed as desolvation gas at 300°C and a flow rate of 8 L/min. The nebulizer was set to 45 psig. The Sheat gas temperature was set at 400°C and a flow of 12 L/min. A potential of 3.5 kV was used on the capillary for positive ion mode. The fragmentor was set to 175 V. MS spectra were recorded in the 150 to 1000 *m/z* range. Metabolite identification was accomplished by means of HRMS data and comparison with the Metlin database (Scripps Center for Metabolomics, https://metlin.scripps.edu). Two independent replicates were analyzed.

### Gel zymography.

The protocol used for detection of secreted proteases in spent media derived from Salamone et al. (96) with minor modifications. Tris-glycine SDS-PAGE gels were prepared (97) adding, just in the resolving gel, a warmed gelatin (Gelatin from bovine skin, Sigma-Aldrich) solution to get a final concentration of 0.54 g/L. Gelatin was used as the substrate to detect the presence of proteases. All isolates were grown in LB or FermII (20 g/L dextrin, 10 g/L tryptone, 1 g/L KH_2_PO_4_, 3.4 g/L K_2_HPO_4_, 0.3 g/L MgSO_4_ · 7H_2_O, 0.01 g/L FeSO_4_ · 7H_2_O, 0.1 g/L ZnCl_2_, 0.01 g/L CuSO_4_ · 7H_2_O, 0.003 g/L MgCl_2_ · 4H_2_O, 0.01 g/L CaCl_2_, 0.03 g/L NaCl, pH 7) (98) at 30°C in shaking conditions for 1 to 5 days to have rich cultures. Then, 10 μL of spent medium obtained after centrifugation were added to 10 μL of 2× native loading buffer (50 mM Tris-HCl pH 6.8, 0.1% wt/vol bromophenol blue, 10% vol/vol glycerol, 10% wt/vol SDS) and loaded in gels. After electrophoresis, gels were first washed for 10 min at room temperature and under shaking with wash buffer (2.5% vol/vol Triton X-100, 0.02% wt/vol NaN_3_) to remove running buffer traces, then they were incubated in activation buffer (1.5% vol/vol Triton X-100, 0.02% wt/vol NaN_3_, 2 mM CaCl_2_ · 2H_2_O, 50 mM Tris-HCl pH 7.5) for 1 h at 50°C to activate proteases. Gels were stained with a staining solution (1 g/L Coomassie brilliant blue R-250, 10% vol/vol acetic acid, 50% vol/vol MeOH) overnight under gentle shaking. Finally, gels were treated with a destaining solution (7.5% vol/vol acetic acid, 5% vol/vol MeOH) and presence of proteases was assessed as the appearance of clear bands on the stained background.

### Data availability.

Shotgun sequencing reads have been deposited in the Sequence Read Archive (SRA) database and are available under the BioProject with accession number PRJNA877812.

Draft genome was deposited at DDBJ/ENA/GenBank under the accession JAOAQK000000000. The version described in this paper is version JAOAQK010000000. The BioProject accession number is PRJNA877812.
