# Supplementary material for: Novel Sources of Biodiversity and Biomolecules from Bacteria Isolated from a High Middle Ages Soil Sample in Palermo (Sicily, Italy)
Source: Microbiol Spectr. 2023 Apr 18;11(3):e04374-22. doi: 10.1128/spectrum.04374-22 (PMC10269861; doi:10.1128/spectrum.04374-22)
Supplement: Supplemental file 1 — Fig. S1 to S3 and Tables S2 and S5. Download spectrum.04374-22-s0001.pdf, PDF file, 0.8 MB [file spectrum.04374-22-s0001.pdf]

Supplemental materials

Figure S1. Antibiotic activity of methanolic extracts of *Streptomyces* sp. AV19 against *K. rhizophila*. 5 and 10 μL of the extract were spotted. 10 μL of methanol were spotted as negative control.

Figure S2. HRMS spectum of aureothin, from methanolic extract of *Streptomyces* sp. AV19, containing $[M+H]^+$ and $[M+Na]^+$ adducts.

Figure S3. Phylogenetic tree based on the complete 16S rDNA sequence of *Streptomyces* sp. AV19. Bootstrap values are reported.

Table S1. Top 10 BLAST hits. Alignments were performed using the 16S rDNA of each isolate as query and BLAST's nucleotide collection as database.

Table S2. DNA extraction data.

Table S3. Bracken raw output. Each detected species is reported in association with the NCBI taxonomic ID, the number of raw reads (num), and relative abundance (frac) for each of the two applied extraction protocols.

Table S4. Validation of ancient DNA sequences. The deamination level is reported for both 5'- and 3'-end for both extraction protocols. The delta value indicates the edit distance with respect to the reference genome. The number of ancient and total reads associate to each species was reported.

Table S5. Diameters of inhibition halos. Measures derived from three independent replicates and average diameters are reported in mm with the corresponding standard deviation.

Table S6. Secondary metabolite biosynthetic gene clusters present in genome of *Streptomyces* sp. AV19 and predicted using antiSMASH.

Table S7. Species of whorl-forming *Streptomyces* used for the 16S rDNA-based phylogenetic analysis.

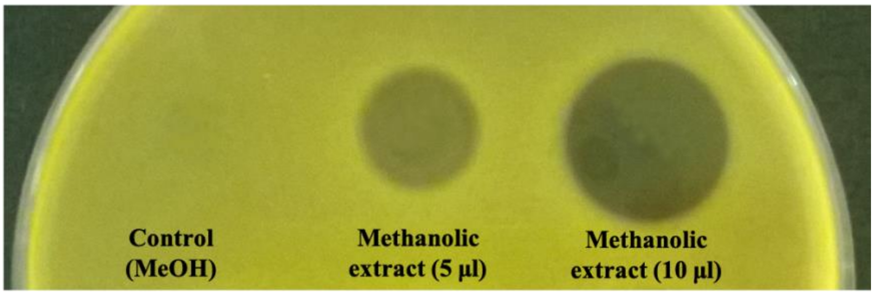

Figure S1. Antibiotic activity of methanolic extracts of *Streptomyces* sp. AV19 against *K. rhizophila*. 5 and 10 µL of the extract were spotted. 10 µL of methanol were spotted as negative control.

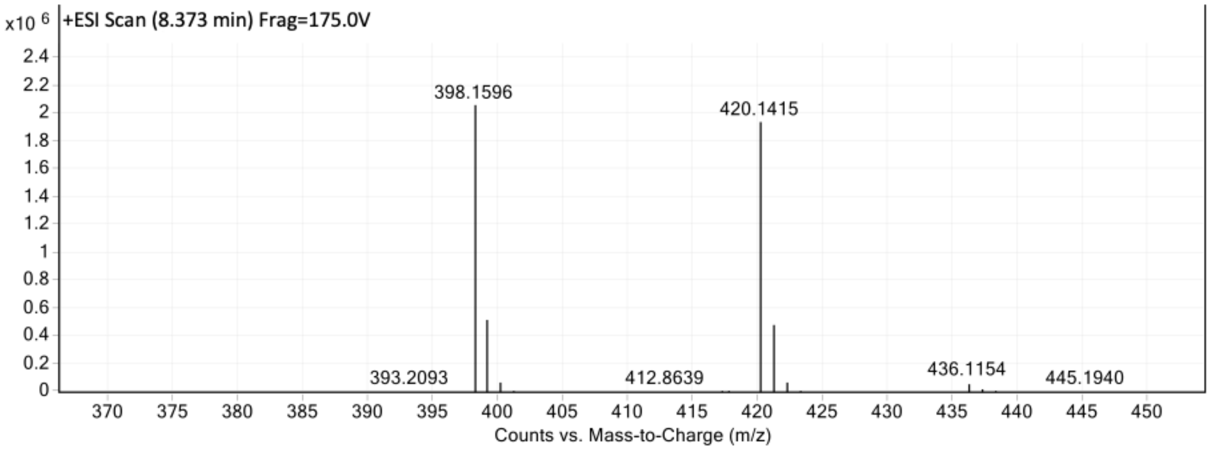

Figure S2. HRMS spectum of aureothin, from methanolic extract of *Streptomyces* sp. AV19, containing $[M+H]^+$ and $[M+Na]^+$ adducts.

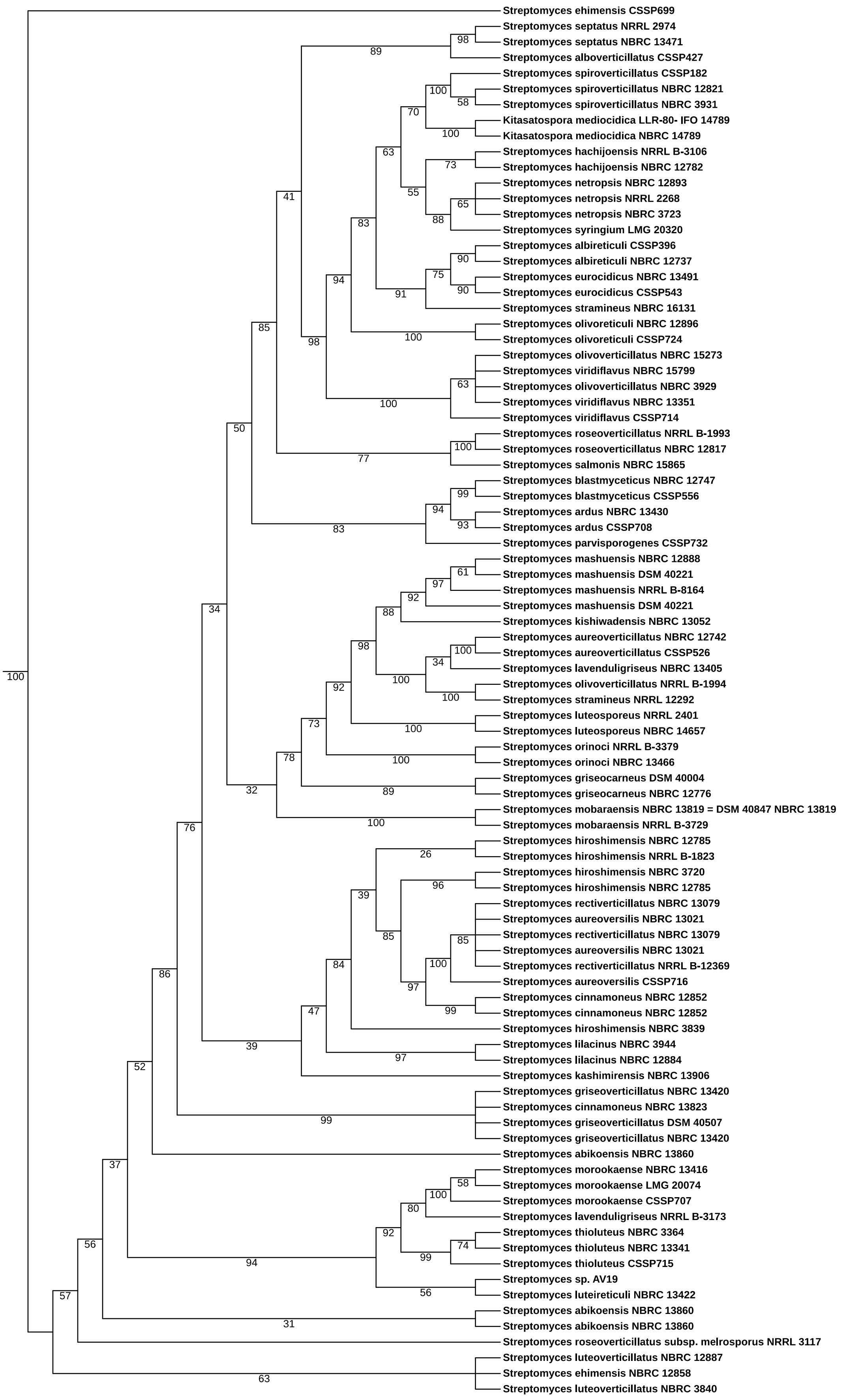

Figure S3. Phylogenetic tree based on the complete 16S rDNA sequence of *Streptomyces* sp. AV19. Bootstrap values are reported.

Table S2. DNA extraction data.

Extraction Method	Sample (mg)	Yield (ng/µL)	Normalized Yield (ng/mg)
**D**	50	2.44*	2.93
**PS**	200	1.49*	0.45

*final elution into 60 µL

Table S5. Diameters of inhibition halos. Measures derived from three independent replicates and average diameters are reported in mm with the corresponding standard deviation.

		Targets		
	Medium	*E. coli*	*K. rhizophila*	*S. cerevisiae*
**8**	R2YED	21 ± 1	50.3 ± 0.6	25.7 ± 0.6
	LB	32.7 ± 1.2	46.3 ± 1.5	28 ± 1
	MS	18.3 ± 1.5	41.7 ± 0.6	14.7 ± 1.5
**19**	R2YED	23.7 ± 0.6	49.7 ± 0.6	27.3 ± 1.2
	LB	29.7 ± 1.5	49.3 ± 1.2	31.3 ± 1.2
	MS	20.3 ± 1.5	43.3 ± 0.6	19.3 ± 0.6
